# Facile Synthesis of Hydrophilic Homo-Polyacrylamides via Cu(0)-Mediated Reversible Deactivation Radical Polymerization

**DOI:** 10.3390/polym13121947

**Published:** 2021-06-11

**Authors:** Fehaid M. Alsubaie, Othman Y. Alothman, Basheer A. Alshammari, Hassan Fouad

**Affiliations:** 1 National Center for Chemical Catalysis Technology, King Abdulaziz City for Science and Technology (KACST), P.O. Box 6068, Riyadh 11442, Saudi Arabia; bshammari@kacst.edu.sa; 2 Department of Chemical Engineering, King Saud University, P.O. Box 800, Riyadh 11421, Saudi Arabia; 3 Applied Medical Science Department CC, King Saud University, P.O. Box 10219, Riyadh 11433, Saudi Arabia; menhfef@ksu.edu.sa; 4 Biomedical Engineering Department, Faculty of Engineering, Helwan University, P.O. Box 11792, Helwan 11731, Egypt

**Keywords:** polydiethylacrylamide, polydimethylacrylamide, controlled polymerization, radical polymerization, water-soluble homopolymers, dispersity

## Abstract

In this work, copper-mediated reversible deactivation radical polymerization (RDRP) of homo-polyacrylamides was conducted in aqueous solutions at 0.0 °C. Various degrees of polymerization (DP = 20, 40, 60, and 80) of well-defined water-soluble homopolymers were targeted. In the absence of any significant undesirable side reactions, the dispersity of polydiethylacrylamide (PDEA) and polydimethylacrylamide (PDMA) was narrow under controlled polymerization conditions. To accelerate the polymerization rate, disproportionation of copper bromide in the presence of a suitable ligand was performed prior to polymerization. Full conversion of the monomer was confirmed by nuclear magnetic resonance (NMR) analysis. Additionally, the linear evolution of the polymeric chains was established by narrow molecular weight distributions (MWDs). The values of theoretical and experimental number average molecular weights (Mn) were calculated, revealing a good matching and robustness of the system. The effect of decreasing the reaction temperature on the rate of polymerization was also investigated. At temperatures lower than 0.0 °C, the controlled polymerization and the rate of the process were not affected.

## 1. Introduction

Hydrophilic monomers, such as acrylamides, as well as their homopolymers and copolymers, have been utilized in oil recovery, wastewater treatment, and environmental systems [1,2]. The conventional acrylamide polymerization techniques have been widely investigated; however, only few methods utilizing controlled polymerization have been reported. Despite significant advances in controlled polymerization technologies, the reversible deactivation radical polymerization (RDRP) of water-soluble acrylamides remains a challenge [3]. Controlled polymerization in the presence of aqueous media or below ambient temperature is difficult. The fast RDRP in water often leads to undesirable side reactions. To suppress such reactions, controlled polymerization approaches have been employed previously [4,5,6,7,8,9]. 

The recently proposed mechanism of copper-mediated RDRP involved an equilibrium between activating and deactivating species, which was mediated by Cu–ligand complexes [10,11,12,13,14,15]. It has been determined that controlled polymerization of water-soluble acrylamides is a challenging task due to undesirable termination reactions, which lead to the loss of the end group fidelity [16,17,18,19,20,21,22,23]. In the single electron transfer living radical polymerization (SET-LRP) method, which is the latest technique classed as RDRP, the disproportionation of the copper bromide complex in the presence of nitrogen-based ligands occurs to provide the system with Cu(0) catalyst and a CuBr_2_ disruptor species prior to the addition of the initiator and monomer [10]. The disproportionation of Cu(Ι)Br/tris[2-(dimethylamino)ethyl]amine (Me_6_TREN) in an aqueous solution was also examined to prepare multiblock polyacrylamides [20,21]. Equal polymer chain growth (*Ð* < 1.10) and full monomer conversion were achieved using this approach, which was confirmed by nuclear magnetic resonance (NMR) and gel permeation chromatography (GPC) analyses. 

Polydimethylacrylamide (PDMA) is a useful polymer with intriguing properties. It contains a hydrophilic acrylamide group; therefore, it is fully soluble in water and exhibits excellent biocompatibility. It has been extensively used in different industrial applications, e.g., as an associating polymer for controlling the physical behavior of paints and coatings as well as for enhanced oil recovery [24]. The biological applications of PDMA include the preparation of proteins, two-phase catalysts, and drug delivery systems [25,26].

Matyjaszewski et al. [17,18] reported on the first polymerization of *N*,*N*-dimethylacrylamide (DMA) using the aqueous atom transfer radical polymerization (ATRP) method; however, the polymerization was not controlled. Similar results were obtained by Brittain and co-workers [19]. Both groups concluded that controlling the aqueous ATRP of DMA was challenging when only water was used as the solvent at room temperature. Recently, Haddleton and coworkers [21] found that PDMA could be successfully polymerized by aqueous SET-LRP. Although controlled polymerization was conducted, the resulting PDMA displayed a low molecular weight (Mn ~3100 and 5100 g mol^−1^), which could limit the applications of the material in cases where polymeric materials with high Mn are required. 

Reversible deactivation radical polymerization techniques have been developed to become powerful tools for the preparation of well-controlled polymers with predetermined molecular weight and narrow molecular weight distribution. However, mostly, they have still proved problematic in aqueous media. Given the increased interest in water-soluble and biocompatible polymers in advanced applications, this issue in the synthetic toolbox needs to be addressed. In light of the abovementioned issues, the work on them needs more attention. In this work, a facile Cu(0)-mediated RDRP method was utilized to synthesize two well-defined homopolymers containing DMA and diethylacrylamide (DEA) units. Water was used as the solvent to produce the homopolymers below ambient temperature. The polymerization reactions were conducted in a controlled manner to achieve low molecular weight distributions (MWDs). Full monomer consumption was obtained within 1 h, which was evidenced by ^1^H NMR and size-exclusion chromatography (SEC) analyses. In situ CuBr disproportionation was utilized to achieve a rapid synthesis of two different acrylamides. Homopolymerization of both DMA and DEA monomers was performed to afford narrow MWDs. Moreover, we investigated the effect of reducing the temperature of the reaction on the rate of polymerization. Ultimately, the described approach using water as the solvent could be applied both in academic and industrial settings to provide valuable polymers at temperatures below ambient temperature. 

## 2. Materials and Methods

### 2.1. Materials

DEA (99%) and DMA (99%) were supplied by Sigma-Aldrich, Darmstadt, Germany. Prior to use, the monomers were filtered by injection over a column filled with alumina to eliminate inhibitors. High-performance liquid chromatography (HPLC) grade H_2_O (VWR international, LLC, Radnor, PA, USA) was used as the solvent for disproportionation and polymerization processes.

The water-soluble initiator (WSI), namely dihydroxypropane-3-oxy-(2-bromo-2-methylpropionyl), and Me_6_TREN were prepared according to previously reported procedures [27,28]. The latter was stored under nitrogen prior to any experiential work. CuBr (98%, Sigma-Aldrich, St. Louis, MO, USA) was successively washed with acetic acid and ethanol, and then dried under vacuum.

### 2.2. Instruments and Analysis

^1^H NMR spectra were obtained on a 300 and 600 MHz JEOL NMR spectrometer using deuterated solvents (D_2_O) as locking agents. The monomer conversion for the homopolymerization of DMA and DEA was measured by evaluating the integrals of the peaks corresponding to the vinyl protons as well as those attributed to dimethyl and diethyl protons, respectively.

SEC was performed employing the Agilent N29812 system using dimethylformamide (DMF) containing 5 mM NH_4_BF_4_ as the mobile phase at 45 °C. The system was equipped with ultraviolet, refractive index, and viscometer detectors as well as an autosampler, a PLgel 5 μm MIXED-D column (300 × 7.5 mm), and a PLgel 5 μm guard column (50 × 7.5 mm). In addition, an Agilent 1260 infinity ii isocratic pump with a maximum pressure of 600 bar was used.

Linear PMMA standards in the range of 200 to 1.0 × 10^6^ g mol^−1^ were used to calibrate the system of reactions. All samples were passed through a 0.45 μm polytetrafluoroethylene (PTFE) filter. All reactions were conducted under a N_2_ atmosphere employing a typical Schlenk technique. 

### 2.3. Synthesis of PDEA and PDMA Homopolymers by Aqueous Cu(0)-Mediated Polymerization at 0 °C

**Scheme 1:**
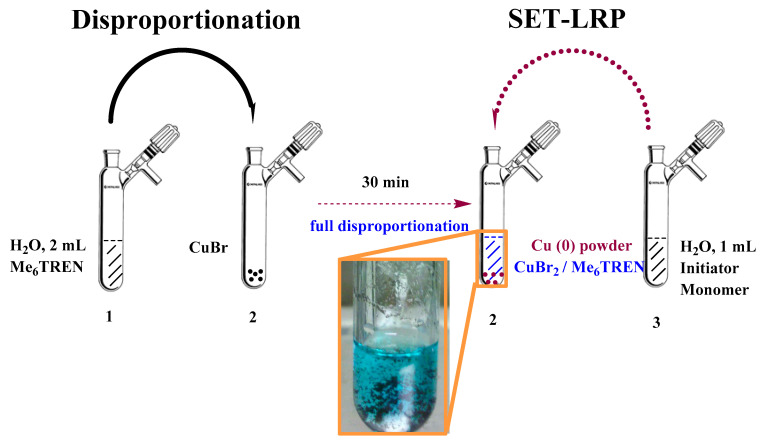
Aqueous Cu(0)-mediated polymerization of acrylamide monomers following a disproportionation reaction.

H_2_O (2 mL), a small magnetic stirrer, and Me_6_TREN-ligand (0.026 mL, 0.0001 mol) were transfer to a Schlenk tube and the solution was bubbled with nitrogen for 15 min (Scheme 1). The degassed solution was transferred to another deoxygenated Schlenk tube containing CuBr (0.014 g, 0.0001 mol) and a small magnetic stirrer via a cannula. The reaction mixture turned blue and red copper particles formed in the solution. The reaction mixture was stirred at 20 °C for 25 min under a N_2_ blanket. Subsequently, the Schlenk tube was placed in an ice-water bath. 

Water (1 mL), dihydroxypropane-3-oxy-(2-bromo-2-methylpropionyl) (0.60 g, 0.00025 mol), and acrylamide monomer were added to a new Schlenk tube equipped with a small magnetic stirrer. The amount of monomer was based on the individual degree of polymerization (DP) of DMA or DEA. The solution was deoxygenated by bubbling with nitrogen for 15 min. The deoxygenated solution was subsequently transferred to a Schlenk tube placed in an ice-water bath via a cannula. The reaction solution was stirred at 0.0 °C for different times.

Samples of the reaction solution were carefully removed by a degassed syringe, for 1H NMR and SEC analysis. Then, the taken sample (50 μL) was exposed to air which stopped catalyst from being active. For 1H NMR evaluation, the samples were simply diluted with D_2_O. However, prior to SEC analysis, the samples were passed through an aluminum oxide column to eliminate any unreactive catalyst.

## 3. Results and Discussion

**Scheme 2:**
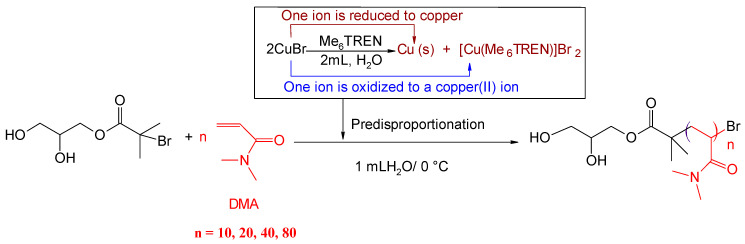
Synthesis of polydimethylacrylamide (PDMA) (degree of polymerization (DP) = 10, 20, 40, and 80) by Cu(0)-mediated polymerization in water at 0.0 °C via prior disproportionation of copper bromide. Conditions: [WSI]:[CuBr]:[Me_6_TREN] = 1:0.4:0.4.

Well-defined homopolymers of two acrylamide derivatives were synthesized via Cu(0)-mediated RDRP in pure water at 0 °C (Scheme 2).

**Figure 1:**
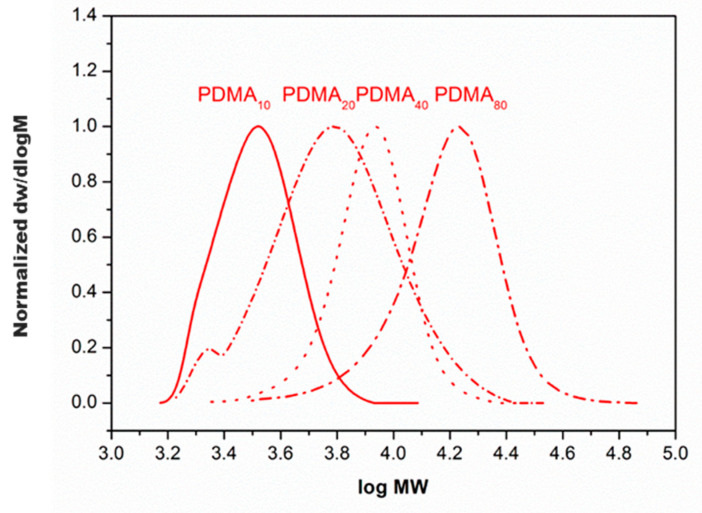
The evolution of molecular weights of PDMA (DP = 10–80) catalyzed by the generated Cu(0) particles/CuBr_2_/Me_6_TREN complex in water determined by gel permeation chromatography (GPC) using dimethylformamide (DMF) as the mobile phase.

Homopolymerization of DMA in aqueous solutions was exploited employing the Cu(0)-mediated polymerization approach. Initially, the polymerization of DMA was investigated using the aforementioned conditions: [DMA]:[WSI]:[CuBr]:[Me_6_TREN] = [10 or 20]:[1]:[0.4]:[0.4]. ^1^H NMR analysis confirmed that 100% monomer conversion was achieved in 8 min for each DP = 10 and 20. SEC traces revealed one symmetrical peak distribution with narrow MWDs (DP = 10, *Đ* ~1.09 and DP = 20, 1.28), demonstrating the controlled nature of the system (Table 1 and Figure 1). 

Nonetheless, the appearance of a small low molecular weight shoulder was detected in the case of DP = 20, indicating the lack of a catalyst. When the target was DP = 40, the synthesis was primarily conducted utilizing previous conditions; however, a broad dispersity (*Đ*) was observed. Thus, the initial catalytic ratio was increased to [copper]:[ligand] = [2]:[1]. The polymerization then reached full conversion within the same reaction time (8 min). Initially, our approach was to fix the initial monomer concentration at 10% *v*/*v* as a workable solution viscosity. Due to the increase in the solution viscosity for the case of DP 80, DMA monomer was dissolved in 4 mL of water instead of 1 mL. Notably, narrow dispersity (*Đ* ~ 1.10) over MWDs was achieved, demonstrating that the polymerization was fast and controlled.

**Figure 2:**
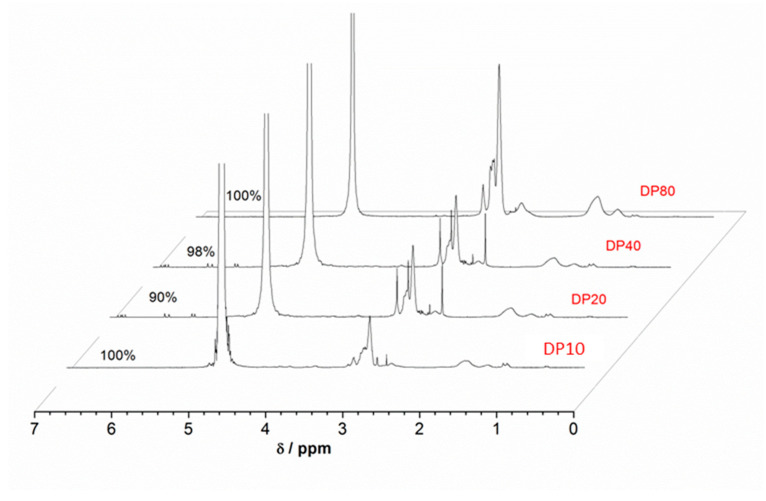
^1^H nuclear magnetic resonance (NMR) spectra of the Cu(0)-mediated polymerization of PDMA (DP = 10, 20, 40 and 80) in D_2_O.

The optimized reaction conditions were used to obtain polymers with high molecular weights (DP = 80). Pleasingly, quantitative conversions and narrow MWDs (~1.19) were achieved within 8 min, clearly demonstrating the remarkable potential of the polymerization processes. A well-defined molar mass polymer (size = 14 K g mol^−1^) was obtained in an ultrafast reaction time (Figure 2 and Table 1). 

We were also interested in the homopolymerization of DEA, which is another intriguing hydrophilic acrylamide. Although PDEA is considered to be biocompatible [29,30] and less toxic than poly(*N*-isopropylacrylamide) (PNIPAM) [31,32], it is considerably less well studied. Both PDEA and PNIPAM exhibit a low critical solution temperature (LCST) in aqueous media (approximately 25–33 °C). Notably, this feature of the polymers can be exploited for specific applications. However, the phase behavior, hydration, and volume transition of PDEA are broader than those of PNIPAM due to the absence of the N–H group [33,34]. This phenomenon is particularly valuable for achieving a progressive release system for encapsulated drugs.

PDEA is less utilized in advanced applications, which can be attributed to the lack of attempts to prepare well-defined PDEA, especially in aqueous media. Delaittre and Charleux [34] were the first to report nitroxide-mediated polymerization (NMP) of DEA in toluene. Gody et al. [22,23] employed reversible addition−fragmentation chain-transfer polymerization (RAFT) to synthesize PDEA; however, the use of a high temperature was required (70 °C). 

**Scheme 3:**
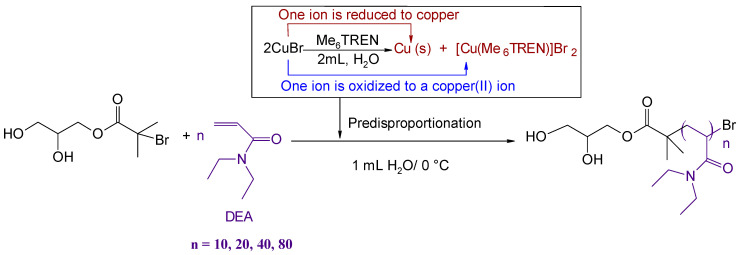
Synthesis of PDEA (DP = 10, 20, 40, 80) by Cu(0)-mediated polymerization in water at 0 °C via prior disproportionation of copper bromide. Conditions: [WSI]:[copper]:[ligand] = 1:0.4:0.4.

**Figure 3:**
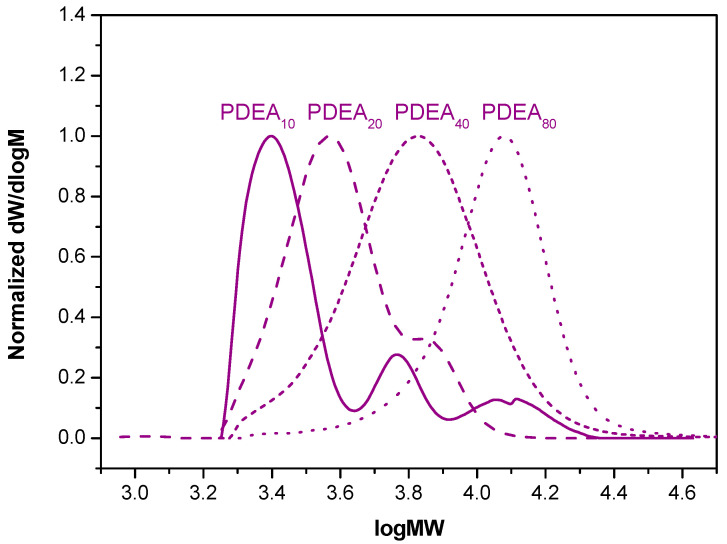
The evolution of molecular weights of PDEA (DP = 10–80) catalyzed by the generated Cu(0) particles/CuBr_2_/Me_6_TREN complex in water determined by GPC using DMF as the mobile phase.

**Figure 4:**
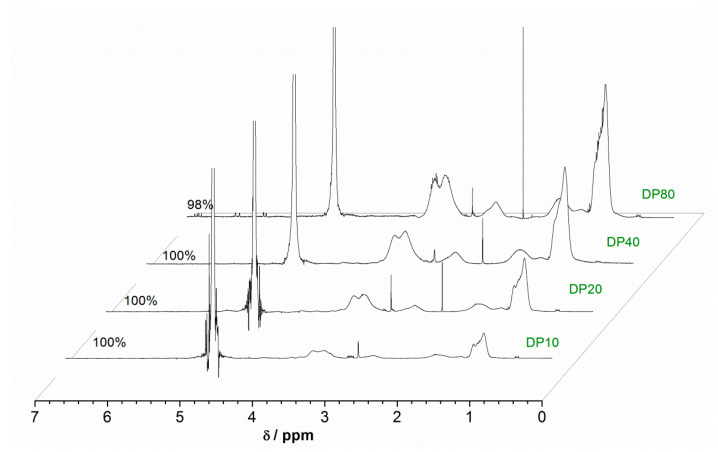
^1^H NMR spectra for Cu(0)-mediated polymerization of PDEA (DP = 10, 20, 40, and 80) in D_2_O.

To investigate the potential of the aqueous copper-mediated RDRP to control the reaction system, four polymerizations, i.e., DP = 20, 40, 60, and 80, were targeted (Scheme 3). Utilizing the previously optimized conditions for PDMA, controlled polymerizations were effectively performed with full monomer consumption. As summarized in Table 2, narrow dispersities were achieved. This was further confirmed by NMR and GPC analyses (Figure 3 and Figure 4). 

Intriguingly, although the reactivity of DEA was similar to that of DMA [35,36,37], compared to the same DP of PDMA, the full conversion of DEA was achieved after a longer reaction time. The GPC evaluation revealed some high molecular weight shoulders for DP = 10 and 20; however, the MWDs were within the controlled limit of less than 1.5. 

### Effect of Decreasing the Reaction Temperature on the Rate of Polymerization

**Figure 5:**
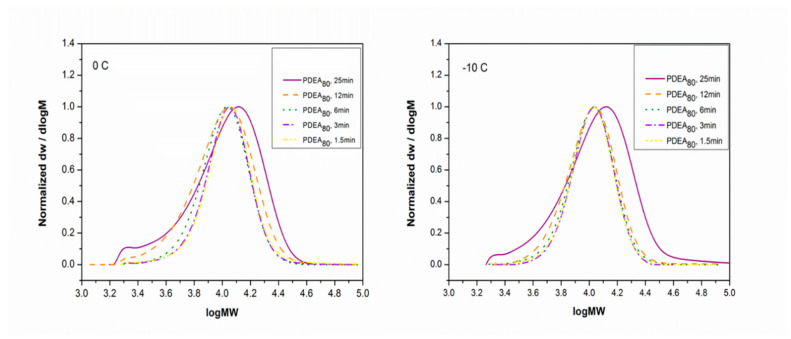
Gel permeation chromatography (GPC) traces showing the molecular weight evolutions for Cu(0)-mediated polymerization of DEA in H_2_O at 0.0 °C and −10 °C.

**Figure 6:**
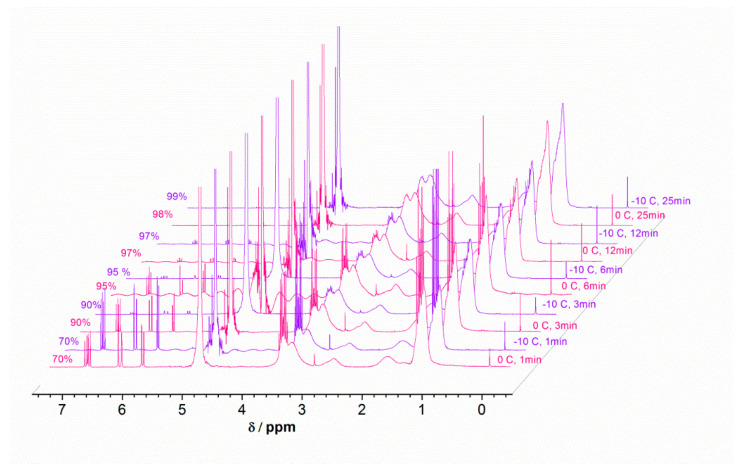
^1^H NMR spectra for Cu(0)-mediated polymerization of DEA in H_2_O at 0.0 °C and −10 °C using the previously mentioned conditions: [WSI]:[copper]:[ligand] in D_2_O.

To investigate the effect of decreasing the reaction temperature on the rate of polymerization, the procedure for the preparation of PDEA was employed. The disproportionation and polymerization conditions were identical to those utilized in experiments involving the acrylamide monomers. DEA (2.72 mL, 20 mmol (DP 80)) was used as a monomer and was allowed to polymerize at 0.0 °C and −10 °C. Two systems were used to decrease the temperature of the reaction. The first consisted of an ice-water bath (0.0 °C), while the second was a mixture of salt and ice (−10 °C). The temperature was monitored at different times using a thermometer. Subsequently, reaction samples taken at specific times were analyzed by ^1^H NMR and SEC. The time and conversion of each polymerization reaction are summarized in Table 3 and Figure 5 and Figure 6.

Notably, 70% conversion was achieved after just 1.5 min, as illustrated by Table 3. The polymerization reaction was controlled and nearly all of the monomer was converted into the polymer after 25 min, demonstrating the effectiveness of the system. Changing the reaction temperature from 0.0 °C to −10 °C did not affect the rate of polymerization, indicating the potential of applying the system in a biological environment. GPC analysis, shown in Figure 5, confirms the occurrence of controlled polymerization. Figure 6 shows the monitoring of monomer conversion by 1H NMR. 

## 4. Conclusions

In this work, syntheses of hydrophilic homopolymers of two acrylamide-based monomers in aqueous media at temperatures below room temperature were successfully conducted. In addition, a facile route for the preparation of discreet PDEA and PDMA in water was developed. Rapid polymerization was achieved after full disproportionation of copper bromide. The occurrence of controlled polymerization was confirmed by GPC analysis, while the monomer conversion was monitored by ^1^H NMR. Employing Cu(0)-mediated RDRP, several PDMA and PDEA polymers (DP = 10, 20, 40, and 80) with excellent MWDs were obtained. Moreover, the results illustrated that the polymerization could be performed at a low temperature (−10 °C) without any decrease in the reaction rate, demonstrating the possible biocompatibility of the developed system. It is confirmed that this versatile technique involving low temperatures can be successfully used to prepare more water-soluble monomers. The synthesis of temperature-responsive copolyacrylamides in aqueous solutions is the main objective of an ongoing study.

## Figures and Tables

**Table 1:** Summary of characteristics of different DPs of dimethylacrylamide (DMA) by Cu(0)-mediated polymerization under various conditions.

DP	H_2_O, mL	Conv. (%)	Time of Polymerization (min)	M_n, th_	M_n, SEC_	*Ð*
10	1	100	8	1200	2900	1.09
20	1	100	8	2200	5400	1.28
40	1	100	8	4200	7900	1.10
80	4	100	8	8200	14,200	1.19

**Table 2:** Summary of characteristics of different DPs of diethylacrylamide (DEA) by Cu(0)-mediated polymerization under various conditions.

DP	H_2_O, mL	Conv. (%)	Time of Polymerization (min)	M_n, th_	M_n, SEC_	*Ð*
10	1	100	25	1500	3100	1.38
20	1	100	40	2800	3800	1.16
40	1	100	40	5300	6000	1.24
80	4	98	80	10,400	10,300	1.19

**Table 3:** Summary of characteristics of polymers of DEA obtained by Cu(0)-mediated polymerization. Conditions: [WSI]:[copper]:[ligand] = 1:0.4:0.4.

Time of DEA Polymerization (min)	Temperature of Reaction	Conv (%)	M_n,th_	M_n,GPC_	*Ð*
1.5	0.0 °C	70	7300	7800	1.18
	−10 °C	72	7300	8100	1.19
3.5	0 °C	90	9400	8600	1.16
	−10 °C	90	9400	8800	1.18
6.5	0 °C	95	9900	10,000	1.16
	−10 °C	95	9900	9800	1.16
12	0.0 °C	97	10,100	10,000	1.16
	−10 °C	97	10,100	10,000	1.16
25	0.0 °C	98	10,400	11,000	1.13
	−10 °C	99	10,400	11,000	1.13

## Data Availability

The data presented in this study are available on request from the corresponding author. The data are not publicly available due to privacy.
